# Characterizing juvenile salmon predation risk during early marine residence

**DOI:** 10.1371/journal.pone.0247241

**Published:** 2021-02-19

**Authors:** Elizabeth M. Phillips, John K. Horne, Jeannette E. Zamon

**Affiliations:** 1 School of Aquatic and Fishery Sciences, University of Washington, Seattle, Washington, United States of America; 2 Fish Ecology Division, Point Adams Research Station, Northwest Fisheries Science Center, NOAA Fisheries, Hammond, Oregon, United States of America; Texas A&M University, UNITED STATES

## Abstract

Predation mortality can influence the distribution and abundance of fish populations. While predation is often assessed using direct observations of prey consumption, potential predation can be predicted from co-occurring predator and prey densities under varying environmental conditions. Juvenile Pacific salmon *Oncorhynchus* spp. (i.e., smolts) from the Columbia River Basin experience elevated mortality during the transition from estuarine to ocean habitat, but a thorough understanding of the role of predation remains incomplete. We used a Holling type II functional response to estimate smolt predation risk based on observations of piscivorous seabirds (sooty shearwater [*Ardenna griseus*] and common murre [*Uria aalge*]) and local densities of alternative prey fish including northern anchovy (*Engraulis mordax*) in Oregon and Washington coastal waters during May and June 2010–2012. We evaluated predation risk relative to the availability of alternative prey and physical factors including turbidity and Columbia River plume area, and compared risk to returns of adult salmon. Seabirds and smolts consistently co-occurred at sampling stations throughout most of the study area (mean = 0.79 ± 0.41, SD), indicating that juvenile salmon are regularly exposed to avian predators during early marine residence. Predation risk for juvenile coho (*Oncorhynchus kisutch*), yearling Chinook salmon (*O*. *tshawytscha*), and subyearling Chinook salmon was on average 70% lower when alternative prey were present. Predation risk was greater in turbid waters, and decreased as water clarity increased. Juvenile coho and yearling Chinook salmon predation risk was lower when river plume surface areas were greater than 15,000 km^2^, while the opposite was estimated for subyearling Chinook salmon. These results suggest that plume area, turbidity, and forage fish abundance near the mouth of the Columbia River, all of which are influenced by river discharge, are useful indicators of potential juvenile salmon mortality that could inform salmonid management.

## Introduction

Assessing predation mortality is an important component of ecological research and resource management, including studies of threatened and endangered species such as Pacific salmon (*Oncorhynchus* spp). Numerous salmonid stocks from the Columbia River Basin on the west coast of North America are experiencing low population abundances and slow recoveries [1]. Mortality of juvenile salmon during the transition from estuarine to ocean habitat has a significant influence on survival to adulthood, and predation is thought to be a primary cause [2–4]. However, a comprehensive evaluation of the spatial distribution of predation risk for different salmon populations during the early marine phase remains incomplete.

An important group of predators near the mouth of the Columbia River are piscivorous seabirds including sooty shearwaters (*Ardenna grisea*) and common murres (*Uria aalge*) that occur in high densities in nearshore Washington and Oregon waters during spring and summer [5–7]. The response of seabirds to prey is often characterized by a Holling type II functional response [8–10], which describes predation rate as an asymptotic relationship of increasing prey density, predator attack rate, and prey handling time [11]. Shearwaters and murres in this region forage on aggregations of small coastal pelagic fish (i.e., forage fish) [12, 13], dominated by species including northern anchovy (*Engraulis mordax*) [14]. Juvenile salmon, referred to as smolts during seaward migration, are similar in length and appearance to forage fish, and become part of the seabird prey community as they migrate from the lower Columbia River estuary to coastal marine habitats. Prey-switching predators, including shearwaters and murres, can vary predation rates when multiple prey species are available and may have significant impacts on less abundant prey [15, 16]. Smolts have been detected in the diets of shearwaters and murres coincident with other prey species [12, 13], suggesting that predation on juvenile salmon during the period of early marine residence may be influenced by the availability of alternative prey [17, 18].

Predation is also influenced by physical and biological processes that affect prey densities and detectability. For example, migration from natal freshwater habitats to the ocean can concentrate smolts in a relatively small area, increasing local densities and potential encounters with co-occurring predators [19, 20]. Turbidity, which increases with river flow [21, 22], can reduce light levels and inhibit the visual foraging range of predatory fish [23–25], which may provide a predation refuge for smolts during downstream migration [26–28]. However, turbid waters near the mouth of the Columbia River attract shearwaters and murres and may increase smolt interactions with avian predators [29]. The survival of Columbia River Basin smolts has also been linked to water temperature and Columbia River plume size [30, 31]. Most smolts occupy nearshore coastal waters during early marine residence [32, 33], and are found in habitats with a narrow range of water temperatures that maximize growth and survival [31]. Variation in Columbia River plume size, as measured by plume volume and surface area [34], has a positive effect on the survival of interior Columbia River Basin subyearling Chinook salmon (*O*. *tshawytscha*) [30]. While numerous factors have been linked to smolt survival, relationships between physical processes, prey densities, and predation mortality have not been quantified.

Smolt predation by avian predators during early marine residence is challenging to assess due to difficulties in obtaining diet samples from seabirds at sea. In the absence of direct observations of predation events, models can be used to predict potential predation, or predation risk [35–37]. We used observations of co-occurrence among seabirds, smolts, forage fish, and the expected Holling type II functional response of shearwaters and murres to develop an index of juvenile salmon predation risk. We assessed how forage fish may mediate predation risk, and evaluated the influence of geographic and physical factors including distance from shore, turbidity, and plume surface area on risk variation and magnitude. Because marine survival of juvenile salmon is often measured by the number of returning adults from a cohort [38, 39], we then compared annual risk estimates to lagged returns of adult coho and Chinook salmon to determine if survival is related to predation risk during early marine residence.

## Materials and methods

All animal work was conducted according to relevant national guidelines. Fish were collected under the Endangered Species Act (ESA) Section 10 permit #1410–7A, which is the federal procedure for research directed by NOAA that includes ESA-listed species.

**Fig 1:**
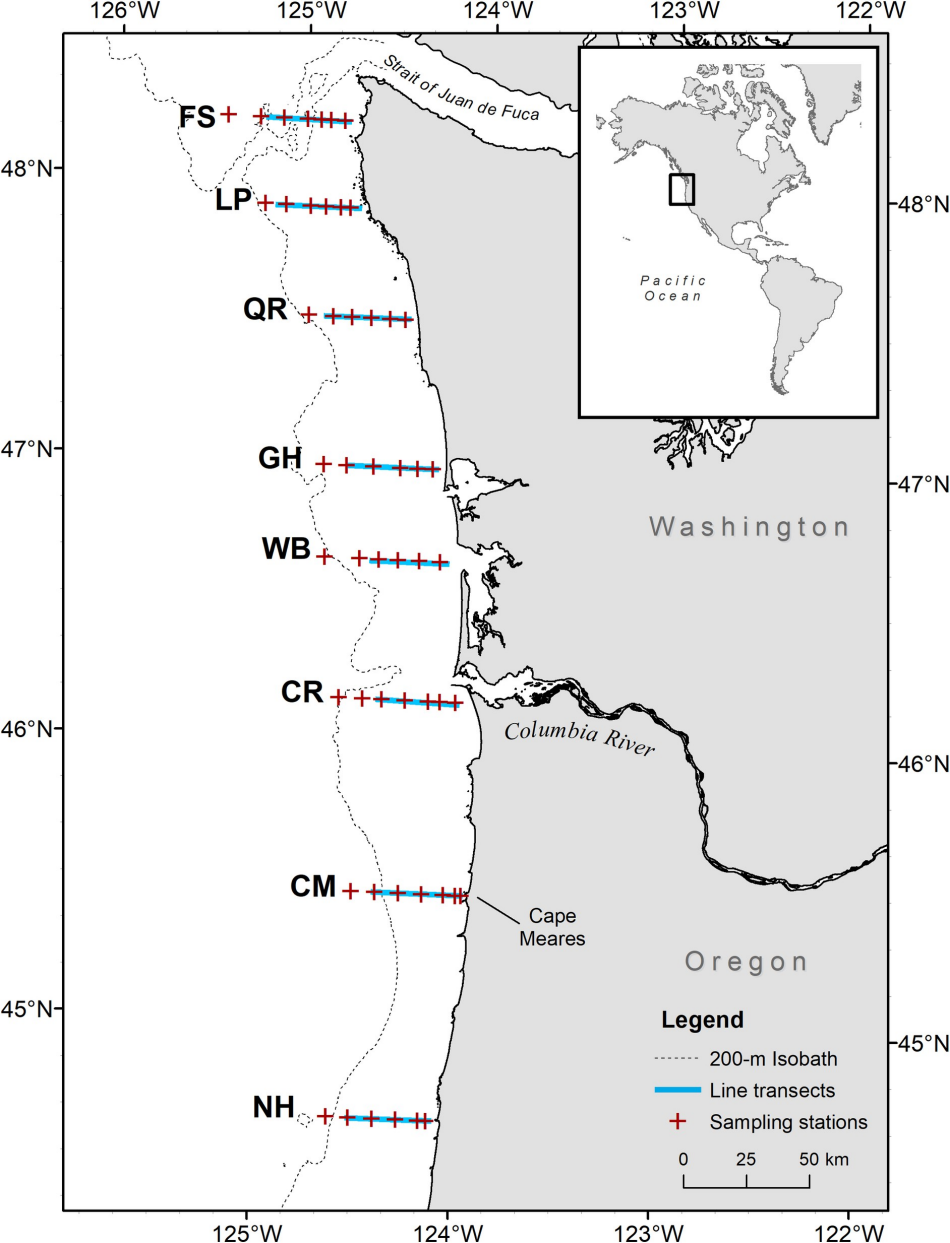
Study area along the Oregon and Washington, USA, coastline. Each transect is named for a geographic feature in proximity to the inshore end of the line as follows: FS, Father and Son; LP, La Push; QR, Queets River; GH, Grays Harbor; WB, Willapa Bay; CR, Columbia River; CM, Cape Meares; NH, Newport Hydrographic.

This study used data from NOAA Fisheries Northwest Fisheries Science Center’s Juvenile Salmon and Ocean Ecosystems Survey (JSOES) research program designed to examine the ocean ecology of juvenile salmon off the Washington and Oregon coasts [40]. Predator, prey, and environmental data were collected during daylight hours in May and June 2010–2012 on chartered commercial fishing vessels sampling along transects and at fixed stations from the central Oregon coast (Newport) to the northern coast of Washington State. We used data from 198 stations sampled on 42 transects during six surveys (Fig 1).

### Predator and prey sampling

Seabirds were counted along transects that began 35–42 km offshore at dawn with the vessel traveling inshore (due east) for 2 h at ~5 m s^-1^ using standard strip transect survey methods (Fig 1) [41]. We recorded all seabirds that were flying or sitting on the water surface, but only used data on shearwaters and murres that were observed floating on the water surface, as birds on the water were assumed to be more closely associated with prey sampled at trawl stations. We estimated densities of sitting shearwaters and murres in 0.09 km^2^ (300 m x 300 m) strips along the starboard side of the survey track.

After seabird counts were complete, the vessel reversed course to collect environmental and fish samples from 5 to 8 fixed sampling stations along the same transect, moving from inshore to offshore. Each station was spaced approximately 9 km apart along the transect (Fig 1). At each station, a profiling conductivity-temperature-depth instrument (hereafter, CTD; SBE 19plus; Sea-Bird Electronics Inc., Bellevue, Washington, USA; http://www.seabird.com/) was deployed to within 5 m of the bottom, or to a maximum depth of 200 m, to record temperature, salinity, and water clarity (measured by % beam transmittance). A 108 m long Nordic 264 rope trawl equipped with 3.0 m Lite® trawl doors (NET Systems Inc., Bainbridge Island, Washington, USA; http://www.net-sys.com/) was fished at the surface for 30 min at a rate of 1.5 m s^-1^ to collect pelagic organisms in the upper 15–20 m of the water column. The net opening was ~30 x 20 m (width x depth) when fishing. All juvenile salmon caught in the trawl were identified and measured to the nearest millimeter (fork length, FL). All non-salmonid organisms were also identified and enumerated, and up to 50 individuals of each species were measured, including fish (FL or standard length, SL) and squid (dorsal mantle length, DML). In instances where more than 50 individuals of a non-salmonid species were caught (e.g., large haul of anchovy), abundance was estimated by weighing a subsample of the catch, counting the subsample, and extrapolating total count based on weight. Trawl catches were standardized to fish km^-2^ by dividing the number of fish caught by area fished. Area fished was calculated as the distance between the start and end of the trawl using GPS coordinates (mean: 3.3 ± 0.7 km, SD), multiplied by the width of the trawl (0.03 km).

To delineate potential prey of seabirds from the rest of the trawl contents, fish catches from each trawl were categorized using known species and species groups consumed by shearwaters [42, 43] and murres [13, 44]. All potential non-salmonid seabird prey items were categorized and grouped as alternative prey, including northern anchovy, Pacific sardine, Pacific herring (*Clupea pallasii*) and California market squid (*Doryteuthis opalescens*) (for full species list see [29]). To ensure that appropriately sized fish were included as prey and to allow for variation in prey shapes (e.g., body length versus body depth), we excluded organisms greater than 250 mm FL or DML, corresponding to the mean FL plus 2 times the standard deviation reported for Pacific sardine (*Sardinops sagax*) consumed by common murres [44]. We did not use a minimum prey length, as murres and shearwaters consume a range of prey sizes including larval and juvenile life stages of marine organisms [45, 46]. All salmon ≤250 mm FL were categorized as juvenile salmon for calculations of total salmon prey density, including coho (*O*. *kisutch*), Chinook, chum (*O*. *keta*), sockeye (*O*. *nerka*), steelhead (*O*. *mykiss*), and cutthroat trout (*O*. *clarkii*). To account for life history variation in length at ocean entry of Chinook salmon, FL and month of capture were used to classify juvenile Chinook salmon as either subyearling or yearling fish based on length-at-age from scale analysis and tagging studies [47, 48]. To facilitate the calculation of predation risk of the three most commonly caught juvenile salmon groups (coho, yearling Chinook, and subyearling Chinook salmon) [29], we analyzed these three salmon groups separately (S1–S3 Figs).

Prey densities in the water column below the trawl depth were measured acoustically during trawling using EK60 or ES60 echosounders (Simrad, Kongsberg Maritime AS, Norway; http://www.simrad.com/) equipped with hull-mounted, split-beam transducers (7° beamwidths measured at half power points) operating at 38 kHz. We used volume backscattering strength (S_v_; dB re 1 m^–1^ [hereafter dB]; see [49]) to quantify acoustic densities through the water column. Acoustic data were processed using Echoview v 5.4 (http://www.echoview.com/), with the S_v_ threshold set to -60 dB (for full details see [29]). Acoustic data between 0–10 m of the surface were excluded to account for transducer depth (4.25 m) and twice the near-field range of the transducers (5.44 m). To develop an index of acoustically-detected prey available to seabirds, all S_v_ measurements were vertically integrated from 10 m below the surface to 70 m depth, the approximate diving range of sooty shearwaters and common murres [50–52]. Acoustic densities were reported as nautical area scattering coefficients (s_*A*_; m^2^ nmi^–2^) [49], indexed in space and time.

To convert acoustic densities to comparable fish densities sampled by the trawl (i.e., fish km^-2^), s_*A*_ values were converted to fish density (ρ_a_) using [53]:

$$\rho_{a}=\frac{\left(\frac{s_{A}}{4\pi *(1852^{2})}\right)}{\sigma_{bs}}*1x10^{6}$$ (1)

Where σ_bs_ (5.50 x 10^−6^ m^2^) is the estimated backscattering cross section of a 150 mm (FL) northern anchovy at 38 kHz with an estimated target strength (TS) of -52.6 dB. The 150 mm FL was based on the maximum fork length of adult anchovy observed in fishery surveys [14]. TS was calculated using the target-strength to length equation for South African anchovy (*Engraulis capensis*) [54]:

$$TS=20*\log\left({FL}_{cm}\right)-76.10$$ (2)

There was no statistical relationship between alternative prey densities estimated from the surface trawl and density estimates from acoustic backscatter (Spearman’s correlation: *ρ* = -0.012, p = 0.857). Therefore, to quantify the total relative alternative prey density (*A*, fish km^-2^) at each station for the vertical foraging range of shearwaters and murres (i.e., ≤ 70 m water depth), non-salmonid prey densities from trawl sampling were summed with densities of acoustically detected alternative prey (S4 Fig). Alternative prey density estimates for May 2010 only include surface trawl catches because acoustic backscatter data were not collected during that survey. Estimates of juvenile salmon density (*S*, fish km^-2^) were not calculated from acoustic samples because smolts typically occur in the upper 10 m of the water column during the day [55, 56], and this near-surface portion of the water column was not sampled acoustically.

### Predator-prey co-occurrence and encounter rate

Predator-prey interactions depend on the co-occurrence of predators with prey at a specific time and place, which increases the probability of a predator encountering, attacking, and consuming prey [57, 58]. In this study, co-occurrence is defined as either shearwaters or murres being present at a station where juvenile salmon were caught in the surface trawl. To quantify co-occurrence, each seabird observation was assigned to the nearest trawl station, using one half the distance between stations as the breakpoint. Because shearwaters and murres consume similar prey and exhibit comparable foraging habits [13, 43, 59], both species were combined in a single predator group for analyses (S5 Fig). To determine areas with consistent predator-prey co-occurrence, we classified stations where seabirds and juvenile salmon co-occurred from those where they did not. Mean co-occurrence was then calculated as the number of times seabirds and juvenile salmon were both present at a station divided by the number of times the station was surveyed. Encounter rate was assumed to be proportional to co-occurrence, based on the observed relationship between foraging seabirds and co-occurring fish schools [e.g., 60].

### Estimating predation risk

We used the Holling type II functional response [11] to relate potential predation upon juvenile salmon (*C*) to the density of co-occurring prey:

$$C=\frac{aN}{1+ahN}$$ (3)

where *N* is prey density, *a* is the combined predator attack and consumption rate (i.e., rate of successful predation), and *h* is prey handling time.

Because juvenile salmon are often consumed coincident with other prey items [16], we assumed equal consumption probabilities for juvenile salmon and alternative prey and hypothesized that potential juvenile salmon predation varies with overall prey density available to predators. To estimate prey density available to predators at each station, we summed all juvenile salmon densities (*S*, fish km^-2^) and alternative prey densities (*A*, fish km^-2^), and used these values to parameterize *N* (fish km^-2^). To calculate the proportion of juvenile coho, yearling Chinook, or subyearling Chinook salmon at each station, we divided the density of fish from each of the three salmon groups (*s*_*i*_) by the total prey density (*N*). This allowed us to estimate potential predation of juvenile coho, yearling Chinook, or subyearling Chinook salmon (*J*_*i*_, smolts consumed time^-1^) as separate components of total potential prey consumption:

$$J_{i}=\left(\frac{aN}{1+ahN}\right)\times \frac{s_{i}}{N}$$ (4)

Seabird attack and consumption rate (*a*, fish time^-1^) was set to 0.6, based on *in situ* observations of seabirds successfully attacking and consuming fish [8, 9]. Handling time (*h*, time) was assumed to be low, based on *in situ* observations of seabirds consuming fish within seconds of capture [8, 9], and was conservatively set to 0.1.

Predation pressure (*P*) was used as a scalar based on seabird density (birds km^-2^), calculated as the total number of shearwaters and murres observed at each station divided by the area surveyed. Predation risk (*R*_*i*_) experienced by individual juvenile coho, yearling Chinook, and subyearling Chinook salmon was expected to increase with greater predation pressure and decrease with greater total densities of smolts (*S*). Thus, to estimate predation risk (*R*_*i*_), potential smolt predation (*J*_*i*_) was multiplied by predation pressure (*P*) and divided by total juvenile salmon density (*S*) at each station:

$$R_{i}=\frac{J_{i}\times P}{S}$$ (5)

Predation risk was calculated separately for juvenile coho, yearling Chinook, and subyearling Chinook salmon at each sampling station for each of the six surveys, resulting in unique, spatially-indexed risk estimates.

### Variation in risk

We compared spatially-indexed predation risk estimates across stations and surveys among the three salmonid groups using a Kruskal-Wallis test [61]. Persistent high-risk areas (i.e., risk hotspots) for juvenile coho, yearling Chinook, and subyearling Chinook salmon were identified by calculating mean predation risk at each station across all surveys. Spatiotemporal variation in risk was visualized using kernel density estimation in ArcMap 10.3 (ESRI, Redlands, CA).

To estimate the change in risk when alternative prey were absent, we re-calculated potential smolt consumption (*J*_*i*_) using only estimates of juvenile salmon density, and then re-calculated predation risk (*R*_*i*_) based on these estimates. Differences in risk estimates with and without alternative prey were compared using a Mann-Whitney rank sum test [61].

The influence of environmental factors on predation risk was evaluated using generalized additive mixed models (GAMMs) with a negative binomial error structure and log link function with the ‘mgcv’ package [62] in R version 3.3.2 [63]. Sampling station was included as a random effect to account for spatial autocorrelation. GAMMs with predation risk as the response variable were parameterized separately for juvenile coho, yearling Chinook, and subyearling Chinook salmon. Covariates in each GAMM included *in situ* measures of turbidity measured by water clarity (% beam transmittance) at 3 m depth sampled during each survey; latitude; distance from shore (km); and contemporaneous daily plume surface area (km^2^) estimated by a hydrodynamic model of Columbia River plume circulation, using salinity values of 28 practical salinity units (psu) to define the plume boundary (Center for Coastal Margin Observation and Prediction; db33 climatological atlas; http://www.stccmop.org/datamart/virtualcolumbiariver). We did not include salinity or temperature as covariates because a correlation matrix indicated that these variables were collinear with multiple predictors. Thin plate regression splines were used as smoothing functions, and spline shrinkage was used to perform automatic smoothness selection of covariates [64]. Backward variable selection was accomplished by first fitting models with all explanatory variables, and removing non-significant terms (p-value > 0.05). Model performance was evaluated by examining deviance explained, changes in Akaike information criterion for small sample sizes (AIC_*c*_), and Akaike weights (*ω*_*i*_). Final models were selected as those with ΔAIC_*c*_ < 2 and *ω*_*i*_ > 0.5. Normalized residuals were plotted to check for violations of model assumptions [65]. The partial effect of each covariate retained in each final model was plotted to examine the relationship between risk and individual physical factors.

The relationship between predation risk during early marine residence and salmon survival was evaluated across the three years by plotting mean predation risk of juvenile coho, yearling Chinook, and subyearling Chinook salmon against adult returns. Returning adult spring and fall Chinook salmon, represented by counts of fish from the corresponding juvenile year class at Bonneville Dam (the first dam on the Columbia River [river km 235] that salmon must pass during their return migration) were obtained from Columbia Basin Research DART data server (www.cbr.washington.edu/dart/adult_annual.html). The majority of yearling Chinook salmon spend two years in the ocean prior to returning to natal rivers in the spring [66], so adult spring Chinook run counts were lagged by two years (i.e., adult spring Chinook salmon returning in 2012 were assumed to represent fish that entered the ocean during 2010). The majority of subyearling Chinook salmon spend three years at sea and return during the fall [67], so fall Chinook salmon run counts were lagged by three years. Adult coho typically return after one year at sea [68], and production is primarily below Bonneville Dam. Therefore, we used adult coho salmon returns to public hatcheries (Oregon Production Index Hatchery, OPIH) reported by the Pacific Fisheries Management Council [69], where ocean survival is estimated as the ratio of hatchery smolt release numbers to hatchery adult freshwater returns in the year following smolt entry into the ocean.

## Results

**Fig 2:**
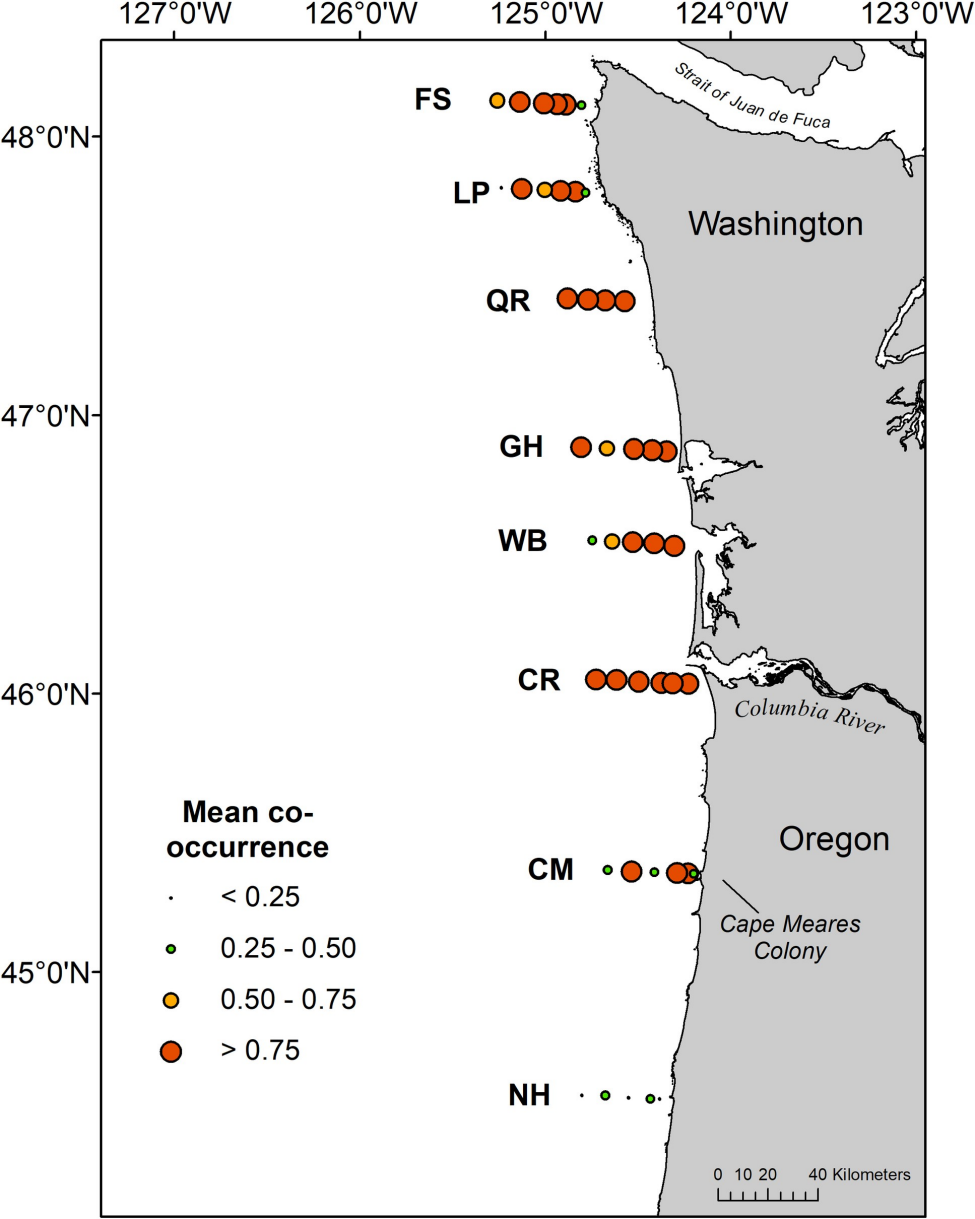
Mean co-occurrence of seabirds and juvenile salmon at sampling stations during May and June 2010–2012. The Cape Meares murre colony identified in the text is labeled.

Frequency of seabird co-occurrence with juvenile salmon at individual stations averaged 0.79 ± 0.41 (SD) across the six surveys, and was consistently high on all transects (mean > 0.75) except the southernmost transect (NH) where numbers of juvenile salmon were low (Fig 2, S1–S3 Figs). Mean co-occurrence was greater than 0.75 at all sampling stations along the Columbia River transect, and the majority of stations sampled on transects along the Washington coast (Fig 2).

**Fig 3:**
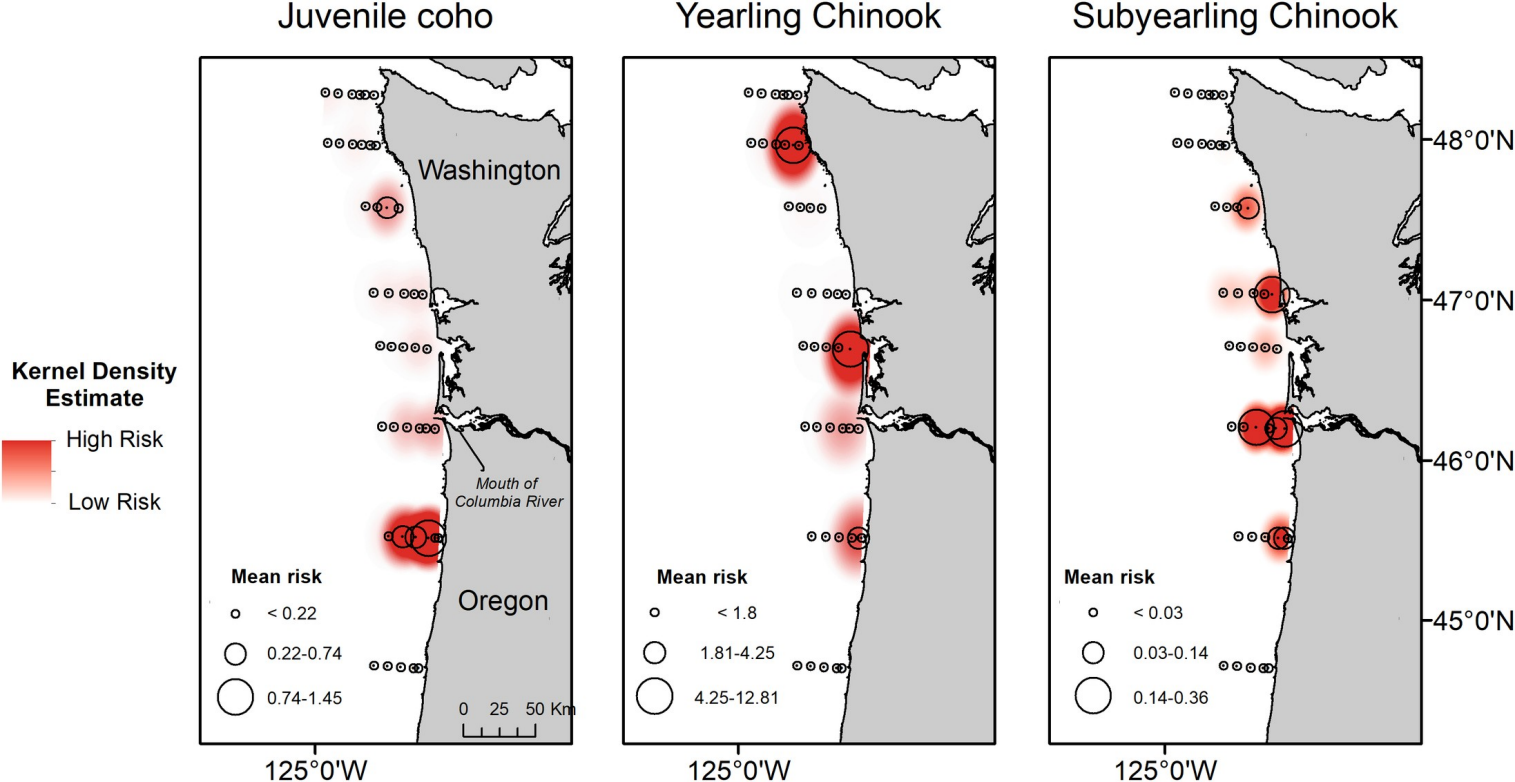
Mean predation risk (R) for juvenile coho, yearling Chinook salmon, and subyearling Chinook salmon sampled at trawl stations during May and June 2010–2012.

Predation risk (*R*) of yearling Chinook salmon (1.08 ± 7.15) was significantly greater than predation risk of coho (0.150 ± 0.77) and subyearling Chinook salmon (0.048 ± 0.20; H_2_ = 50.31, p-value < 0.005). Predation risk for coho was greatest near the Cape Meares transect (Fig 3A), whereas risk estimates for Chinook yearling salmon were greatest near Willapa Bay and La Push (Fig 3B). In comparison, risk for subyearling Chinook salmon was greatest near the mouth of the Columbia River (Fig 3C).

On average, juvenile salmon predation risk dropped by 70.3 ± 3.2% (SD) when densities of alternative prey were included in calculations. Predation risk declined significantly for juvenile coho (-69.0%; W = 16790, p = 0.0092) and yearling Chinook salmon (-68.2%; W = 16144, p = 0.0018). Despite a 73.7% decline in predation risk for subyearling Chinook salmon, the difference was not significant (W = 18912, p = 0.4402). The lack of significance is attributed to the small sample size (n = 54).

**Fig 4:**
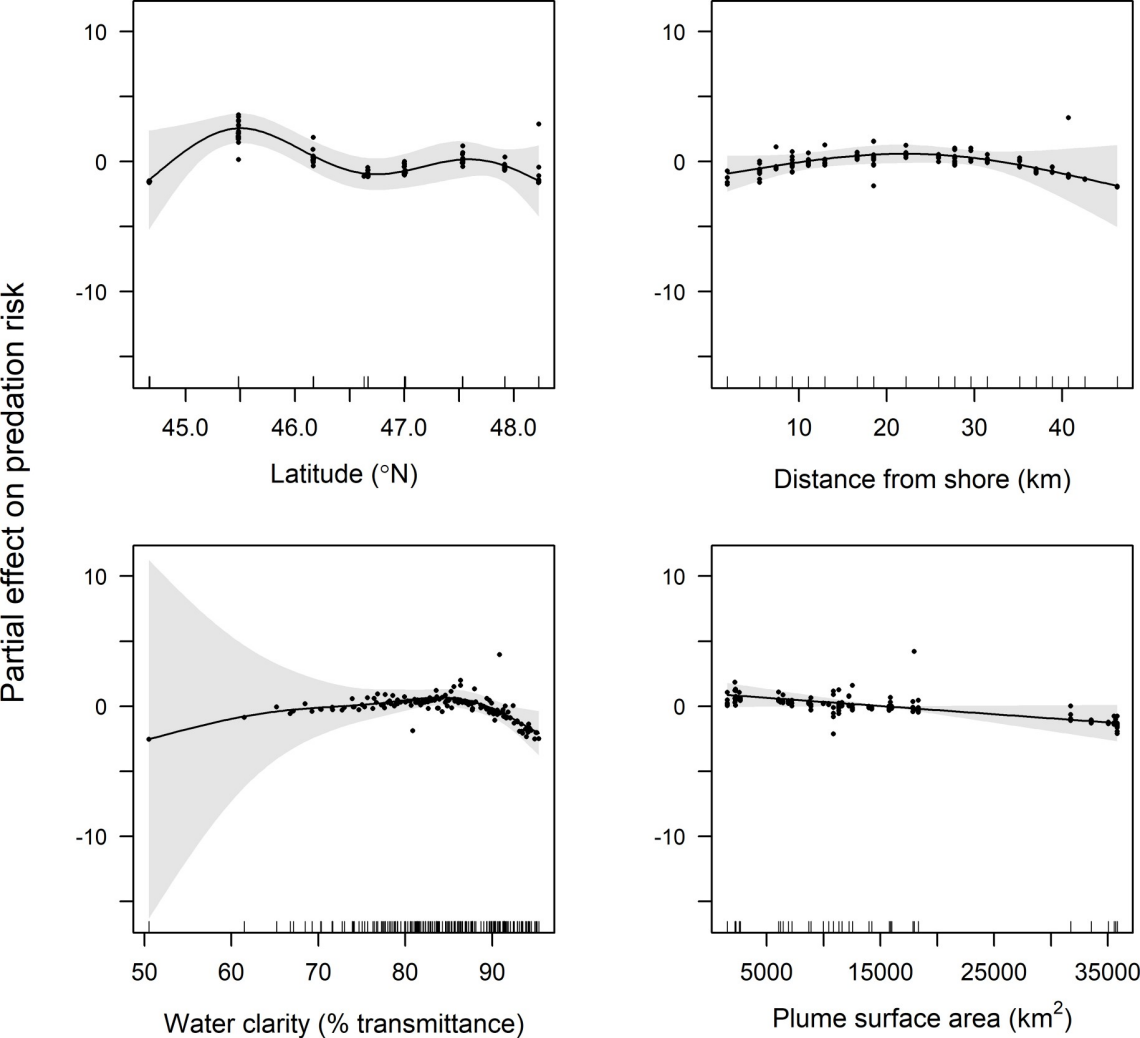
Plots of the partial effects of latitude, distance from shore, water clarity and plume surface area on predation risk of juvenile coho salmon caught in surface trawls during May and June 2010–2012. Points on the plots are partial residuals of the full model without the effect of the term concerned (x-axis covariate). Gray shading around smooth fits represents 95% confidence intervals, and data availability is indicated by tic marks above x-axis.

**Fig 5:**
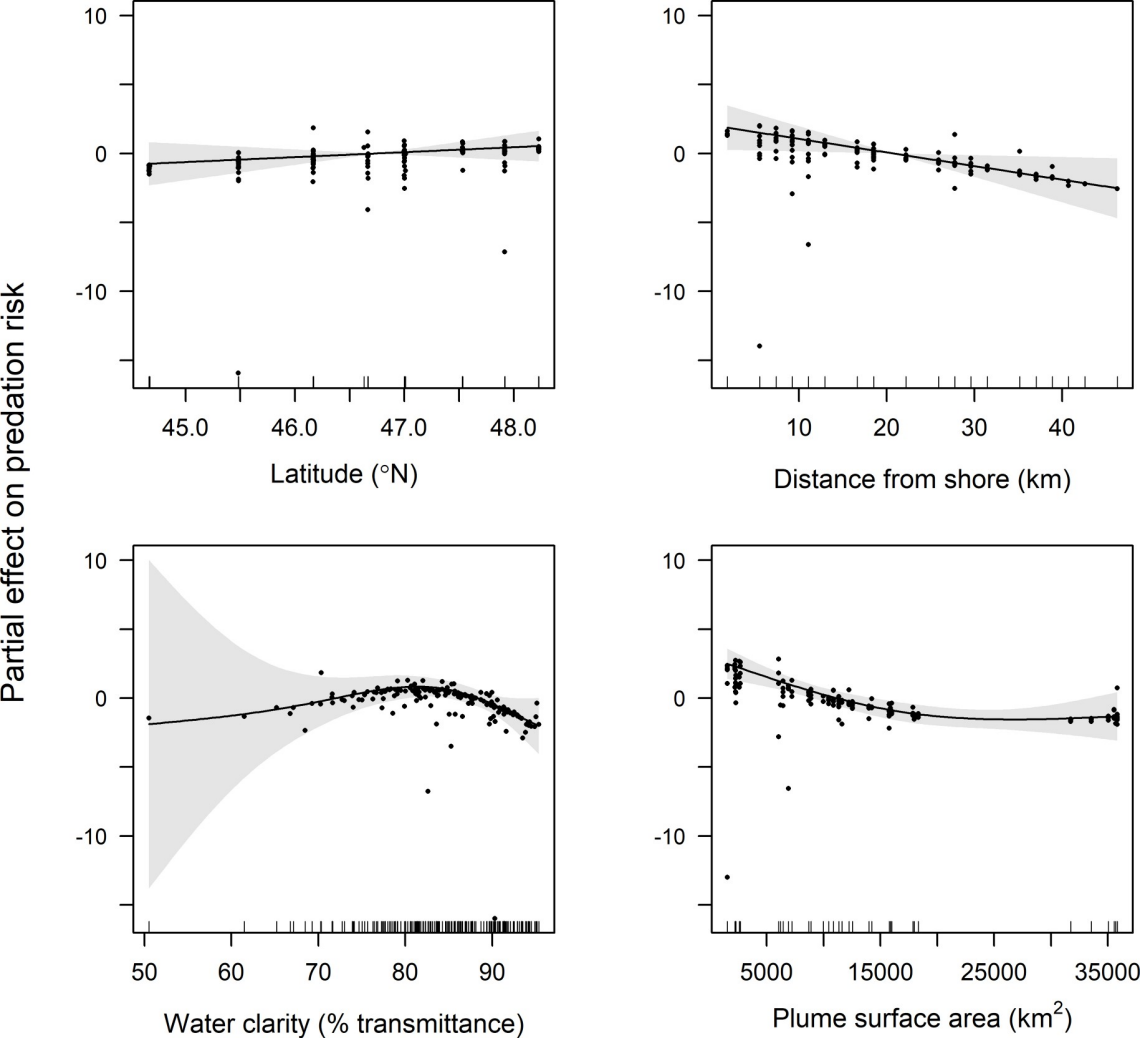
Plots of the partial effects of latitude, distance from shore, water clarity and plume surface area on predation risk of yearling Chinook salmon caught in surface trawls during May and June 2010–2012. Points on the plots are partial residuals of the full model without the effect of the term concerned (x-axis covariate). Gray shading around smooth fits represents 95% confidence intervals, and data availability is indicated by tic marks above x-axis.

**Fig 6:**
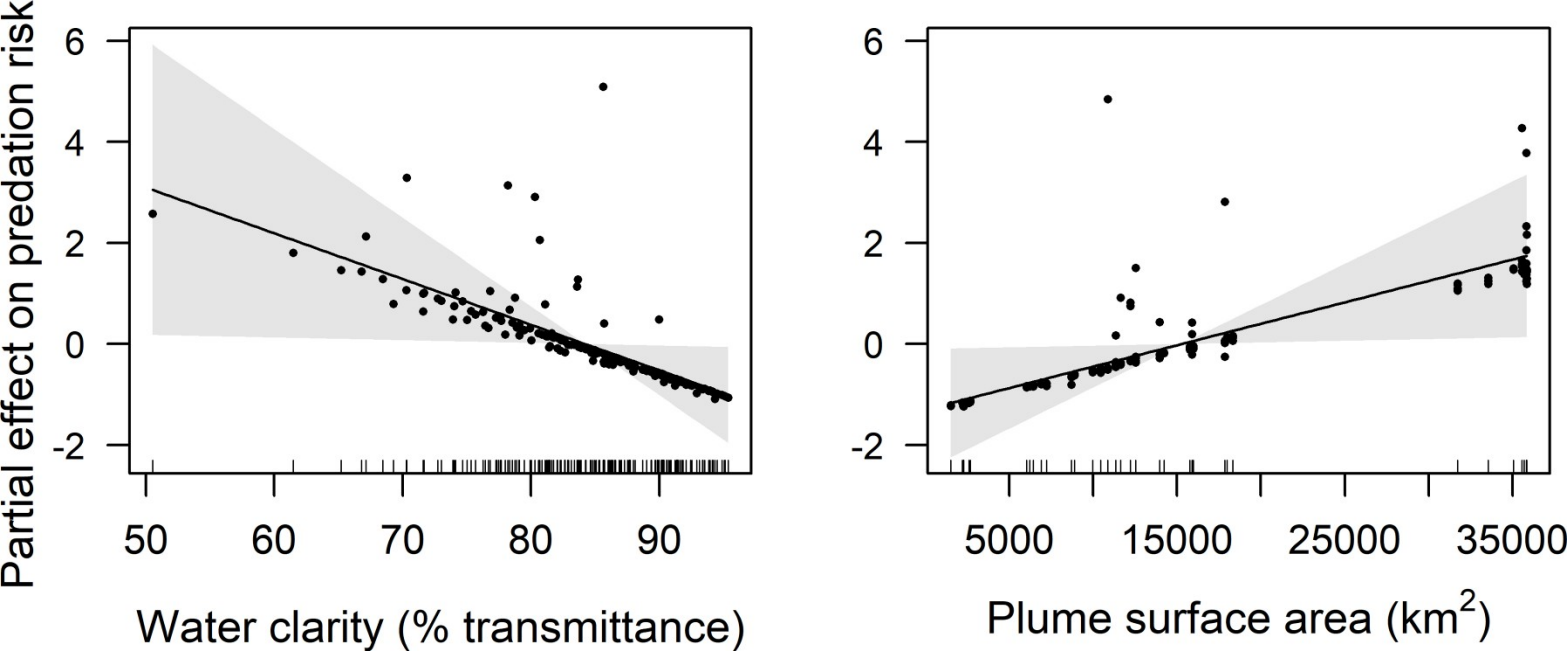
Plots of the partial effects of water clarity and plume surface area on predation risk of subyearling Chinook salmon caught in surface trawls during May and June 2010–2012. Points on the plots are partial residuals of the full model without the effect of the term concerned (x-axis covariate). Gray shading around smooth fits represents 95% confidence intervals, and data availability is indicated by tic marks above x-axis.

GAMM results indicate that latitude, distance from shore, water clarity, and plume surface area were retained in final models of predation risk for juvenile coho and yearling Chinook salmon, while only water clarity and plume surface area were retained in the final model for subyearling Chinook salmon (Table 1). Predation risk for juvenile coho salmon was greatest at around 45.5°N, whereas predation risk increased linearly with latitude for yearling Chinook salmon (Figs 4 and 5). The effect of distance from shore indicated greatest risk for juvenile coho salmon approximately 20 km from shore, whereas risk for yearling Chinook salmon was greatest in nearshore waters (<10 km from shore) and declined across the offshore range sampled (1.9–46.3 km). Predation risk for juvenile coho, yearling Chinook, and subyearling Chinook salmon was greatest in turbid waters, and decreased as waters became more clear (>85% beam transmittance; Figs 4–6). Juvenile coho and yearling Chinook salmon predation risk was highest during periods when plume surface areas were small (< 15,000 km^2^), and declined across the range of surface areas observed (1,535–35,840 km^2^; Figs 4 and 5). In comparison, predation risk of subyearling Chinook salmon was greatest when plume surface areas were large (>15,000 km^2^; Fig 6).

**Table 1:** Generalized additive mixed effects models of predation risk for each salmonid group.

Salmon Group	Model	Description	Formulation	edf	Deviance explained	AIC_*c*_	ΔAIC_*c*_	*ω*_*i*_
Juvenile Coho	**CO.1**	**Full model**	**Risk ~ s(Lat) + s(Distance from shore) + s(Water clarity) + s(Plume area) + s(Station, bs = "re")**	**14**	**49.0**	**121.47**	**0.00**	**0.88**
	CO.2	Drop distance to shore	Risk ~ s(Lat) + s(Water clarity) + s(Plume area) + s(Station, bs = "re")	15	48.3	125.40	3.93	0.12
	CO.3	Drop latitude	Risk ~ s(Water clarity) + s(Plume area) + s(Station, bs = "re")	20	50.5	136.01	14.54	0.00
	CO.4	Drop water clarity	Risk ~ s(Plume Area) + s(Station, bs = "re")	15	38.8	140.08	18.61	0.00
Yearling Chinook	**CY.1**	**Full model**	**Risk ~ s(Lat) + s(Distance from shore) + s(Water clarity) + s(Plume area) + s(Station, bs = "re")**	**23**	**71.8**	**200.60**	**0.00**	**0.72**
	CY.2	Drop latitude	Risk ~ s(Distance from shore) + s(Water clarity) + s(Plume area) + s(Station, bs = "re")	22	70.3	202.49	1.89	0.28
	CY.3	Drop water clarity	Risk ~ s(Distance from shore) + s(Plume area) + s(Station, bs = "re")	18	63.8	222.05	21.46	0.00
	CY.4	Drop distance to shore	Risk ~ s(Plume Area) + s(Station, bs = "re")	23	66.8	227.74	27.14	0.00
Subyearling Chinook	SY.1	Full model	Risk ~ s(Lat) + s(Distance from shore) + s(Water clarity) + s(Plume area) + s(Station, bs = "re")	7	36.1	50.08	3.55	0.09
	SY.2	Drop latitude	Risk ~ s(Distance from shore) + s(Water clarity) + s(Plume area) + s(Station, bs = "re")	5	30.5	47.11	0.57	0.39
	**SY.3**	**Drop distance to shore**	**Risk ~ s(Water clarity) + s(Plume area) + s(Station, bs = "re")**	**4**	**26.7**	**46.54**	**0.00**	**0.52**
	SY.4	Drop water clarity	Risk ~ s(Plume area) + s(Station, bs = "re")	3	11.9	62.21	15.67	0.00

For each model formulation tested, the corresponding effective degrees of freedom (edf), deviance explained, Akaike information criterion corrected for small sample size (AIC_*c*_) and differences (ΔAIC_*c*_), and Akaike weights (ω_*i*_) are presented. Selected final models are in bold.

**Fig 7:**
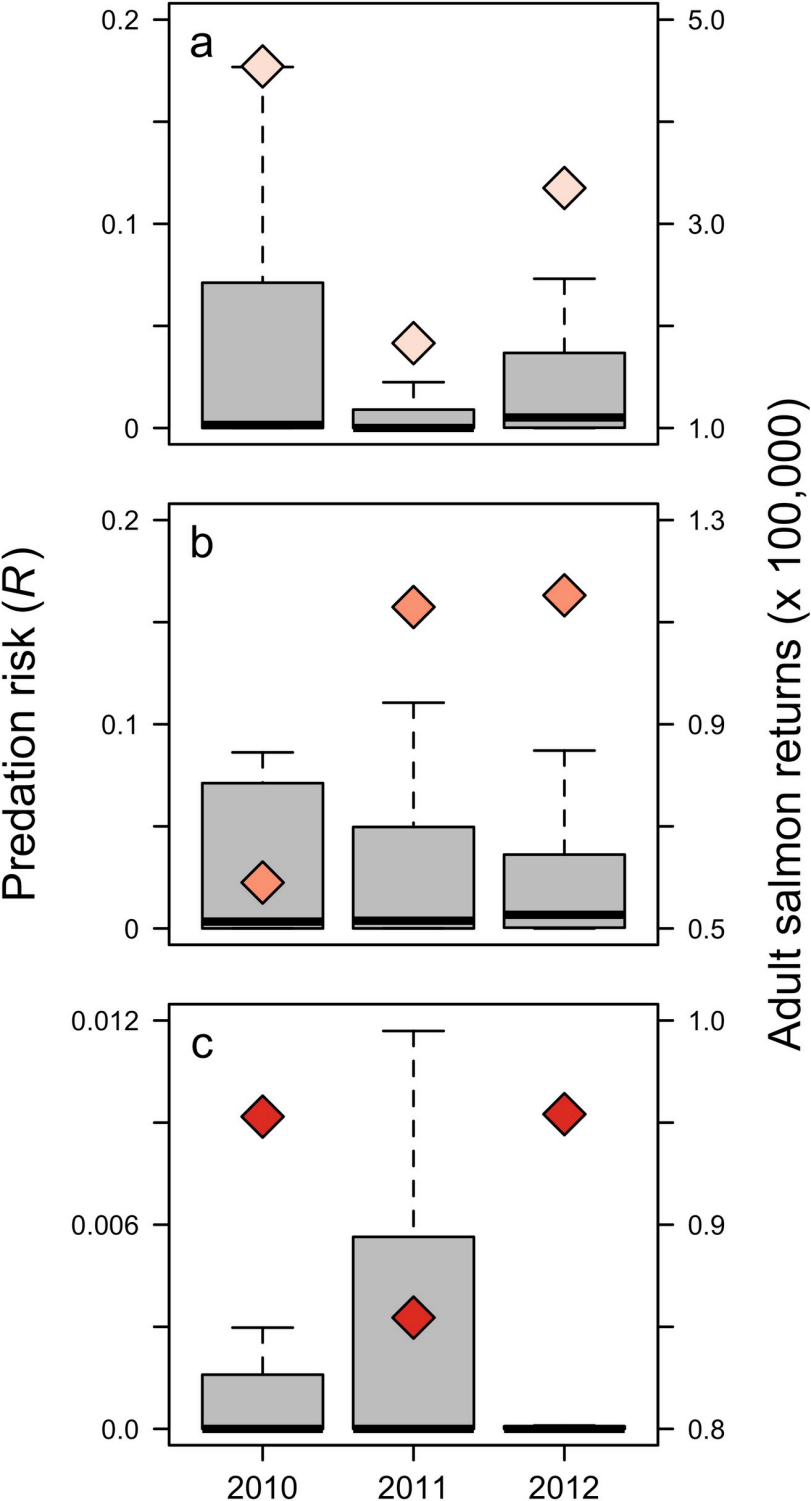
Boxplot of predation risk for (a) juvenile coho salmon, (b) yearling Chinook salmon, and (c) subyearling Chinook salmon. Dark line: median; box: interquartile range (IQR); error bars: max/min within 1.5 x IQR above/below IQR; outliers not shown. Total adult returns (lagged to correspond to predation risk during year of smolt entry) are shown as diamonds.

Annual counts of adult coho salmon returns were positively related to predation risk of juvenile coho salmon (Fig 7A). In contrast, annual counts of adult spring Chinook salmon at Bonneville Dam were negatively related to yearling Chinook salmon predation risk (Fig 7B). Similarly, the relationship between adult returns of fall Chinook and subyearling Chinook salmon predation risk was also negative (Fig 7C).

## Discussion

Using co-occurrence of prey fish and seabirds and a Holling type II functional response, we found that for the three salmon groups examined, juvenile salmon predation risk is greatest in nearshore, turbid waters. Local densities of alternative prey reduced predation pressure on smolts by an average of 70%, indicating that coastal pelagic fish species such as northern anchovy have the potential to influence juvenile salmon mortality during early marine residence [2, 17, 18]. Our results also suggest that a larger Columbia River plume surface area reduces predation risk for juvenile coho and yearling Chinook salmon. Taken together, this study reinforces and expands on findings that the Columbia River plume influences predation pressure experienced by juvenile salmon, and that early marine residence is a period of high mortality risk for ocean-going salmonids [2, 30, 70].

Juvenile salmon co-occurred with seabirds at the majority of sampling stations in this study, indicating that potential encounters between predators and smolts during their seaward migration extends beyond the boundaries of the Columbia River estuary and plume. Smolt-seabird co-occurrence was greatest near the mouth of the Columbia River, which is not surprising given that juvenile salmon emigrate out of the Columbia River to an area where seabirds consistently occur [5–7]. The co-occurrence of smolts and seabirds at sampling stations in the northern portion of the survey area also indicates that juvenile salmon are exposed to avian predators throughout most of their northward migration along the Washington coast. In particular, yearling Chinook salmon may be more vulnerable to predators throughout Washington coastal waters, considering their consistent co-occurrence with seabirds and elevated risk estimates at greater latitudes relative to coho and subyearling Chinook salmon.

Despite consistent co-occurrence of seabirds and smolts throughout most of the study area, we demonstrate that predation risk to juvenile salmon can be reduced substantially when forage fish are available, supporting the alternative prey hypothesis [2, 17, 18]. Aggregation patterns of smolts and alternative prey led to an uneven spatial distribution of predation risk for juvenile coho, yearling Chinook, and subyearling Chinook salmon. Coastal pelagic fish densities are often greatest near the mouth of the Columbia River, and on the Washington coast near Grays Harbor and Willapa Bay [29, 71], where subyearling Chinook and yearling Chinook salmon predation risk was high. Increased risk estimates in this area may be explained by the strong influence of river plume dynamics on salmon and alternative prey densities [29]. Relatively greater predation risk estimates also occurred near Cape Meares for all three salmonids examined, even though co-occurrence estimates were not consistently high on this transect. Cape Meares is the site of a large murre colony [72], and these results suggest juvenile salmon occupying nearshore waters south of the Columbia River mouth [73] may experience greater predation risk in areas where fewer alternative prey occur adjacent to a seabird colony. Similarly, yearling Chinook salmon predation risk was greater in northern Washington waters near La Push, which may be related to greater densities of smolts and seabirds, and relatively fewer alternative prey.

Even when alternative prey densities are high, salmon may still be vulnerable to predation [16]. Greater densities of forage fish near productive plume waters attract large groups of foraging murres and shearwaters [6, 29], which could explain the increased predation risk to co-occurring smolts in nearshore and turbid waters (i.e., apparent competition) [74]. We assumed that shearwaters and murres do not exhibit prey selectivity, but acknowledge that predation risk estimates may vary in alternate models that include prey preference and switching [75–77]. There are no recent data on food habits of shearwaters and murres in the northern California Current, so knowledge of seabird prey selection when two or more prey co-occur is unknown. We also did not consider interference competition in this study, although seabirds are known to be attracted to aggregations of conspecifics [78–80]. While seabirds are generally thought to benefit from collective foraging [81], competition for prey at very high predator densities may alter predation rates [82, 83]. Additional research on seabird encounter, attack, and consumption rates under varying prey and predator community compositions, distributions, and aggregation sizes will expand our understanding of avian impacts on potential prey including juvenile salmon.

Plume surface area is an important factor influencing smolt predation risk, although we found contrasting relationships between plume area and smolt life history. Coho and yearling Chinook salmon rear in freshwater for one year before migrating to sea in May and June [84], when plume surface areas are typically at seasonal maxima [85]. We found that as plume surface area increased above 15,000 km^2^, predation risk for both juvenile coho and yearling Chinook salmon decreased, suggesting that migrating to sea during peaks in river discharge may reduce predation mortality. Increasing freshwater discharge enhances overall production of coastal waters [86], thereby increasing local abundances of multiple trophic levels including zooplankton and larval fish that serve as prey for forage fish and juvenile salmon. Northern anchovy densities are positively correlated with increases in river discharge [87], and anchovy aggregate and spawn near Columbia River plume boundaries in spring and summer [88, 89]. Both shearwaters and murres concentrate in the Columbia River plume when surface areas are low [29] and move towards plume boundary waters when surface areas exceed approximately 1,500–4,000 km^2^ [90]. As plume surface areas become larger, shearwaters and murres track the plume boundary waters and appear to expand their foraging area [29, 90]. This movement of seabirds to the plume boundary may be due to enhanced biophysical coupling [91, 92] that increases foraging opportunities. Therefore, greater plume surface areas may lower juvenile coho and yearling Chinook salmon predation risk by increasing the foraging area for seabirds that are attracted to the Columbia River plume [6, 29, 93], and aggregating alternative prey near plume boundaries, where juvenile salmon do not congregate [94].

In contrast to risk estimates for coho and yearling Chinook salmon, predation risk for subyearling Chinook salmon was higher with greater plume surface areas. Subyearling Chinook salmon migrate to sea after residing for only a few months in freshwater and are therefore smaller in size than yearling salmon [84]. Most subyearling Chinook salmon typically enter the ocean later in the summer, when river discharge is often lower [95]. During periods of above average river flows in spring, however, greater densities of subyearling Chinook salmon can be found in the Columbia River plume and surrounding coastal waters, indicating that these smaller fish may not be able to swim against increased outgoing river flow [55]. Subyearling Chinook salmon also occupy nearshore waters near the mouth of the Columbia River longer than juvenile coho and yearling Chinook salmon [33, 48], making them more vulnerable to seabirds foraging near the mouth of the Columbia River. The observed relationship between subyearling Chinook salmon predation risk and plume surface area could also be related to survey timing and small sample sizes, as we only used samples from spring (May and June) and greater numbers of subyearling Chinook salmon are usually caught in fall (September) surveys when they are more abundant in coastal waters [30, 94].

Predation risk was highest for smolts when water clarity was at low or intermediate values, indicating that turbidity does not provide smolts a refuge from avian predators in the ocean. This is not unexpected given that shearwaters and murres are attracted to turbid plume waters [6, 29, 90], but contrasts to studies of juvenile salmon consumption by piscivorous fish in lakes and rivers [26, 28, 96]. Water clarity measurements used in this study represent a mix of suspended sediment and phytoplankton concentrations associated with the nutrient-rich recirculating plume waters, whereas turbidity in freshwater habitats may be driven by different mechanisms. Despite these differences, previous research has found that seabirds can effectively forage in highly turbid freshwater and saltwater habitats [97–99], which suggests that smolts are vulnerable to avian predators when water clarity is low regardless of the habitat type. Turbidity values in the Columbia River plume are typically highest in near-surface waters (< 5 m), with clarity increasing beneath the surface lens of the plume [100]. Thus, the large footprint of the plume may serve as a predictable surface feature for aerial avian predators to locate before pursuing prey in deeper waters [90].

Our comparison of risk to adult salmon returns indicates that potential predation risk experienced by juvenile salmon during early marine residence may relate to adult returns 2 to 3 years later. However, our results were inconsistent; we found a positive relationship between risk and coho returns, in contrast to a negative relationship for subyearling Chinook and yearling Chinook salmon adult returns. This may relate to the interactive effects of freshwater discharge and ocean conditions on salmon survival, or to our use of only three years of aggregated adult salmon data. The relationships between adult returns and predation risk for each salmon group are likely to change with additional years of data. We also assumed that return timing for salmon in this study was concentrated within a single year, although individuals from the same cohort, particularly for Chinook salmon, return between 2 to 6 years after ocean entry [66, 101]. To increase the utility of a survival index, additional effort using a longer timeline for returning adults may elucidate relationships between predation risk and survival to adulthood. A longer time series to calculate stock-specific juvenile salmon predation risk may also increase precision of risk estimates for threatened and endangered species such as upper Columbia River and Snake River wild spring/summer Chinook salmon.

This study demonstrates that predictions of juvenile salmon early marine survival may be informed by knowledge of river discharge, plume surface area, forage fish abundance, and associated estimates of predation risk. Plume surface area is correlated to river discharge [34], and both metrics are available from monitoring stations on the river and from hydrodynamic model outputs [102]. To reduce juvenile salmon predation risk, springtime river flows and spill regimes could be coordinated to manage river discharge so that plume surface areas during downstream smolt migration remain greater than ~15,000 km^2^. Data from the SELFE model (Center for Coastal Margin Observation and Prediction; db33 climatological atlas; http://www.stccmop.org/datamart/virtualcolumbiariver) suggest that this may not be a difficult goal to achieve. While daily average river plume surface areas for the months of April to July during 1999–2016 exceeded 15,000 km^2^ only 7% of time, plume surface areas were greater than 10,000 km^2^ approximately 22% of the time. Even if spill modification is impractical, knowledge of plume surface areas may be a useful proxy index of smolt early marine mortality that could be incorporated into models of adult salmon survival. Further, river discharge or plume surface area could be used by hatchery managers considering varying smolt release timing in an effort to maximize early marine survival of juvenile salmon. Similarly, knowledge of ocean conditions that influence the abundance and distribution of forage fish may also inform estimates of juvenile salmon survival.

This study used predator-prey theory to estimate predation risk of Columbia River salmon and to examine environmental conditions that affect predation risk. Results suggest that predation on juvenile salmon is greatest nearshore, in turbid waters, and when river discharge is relatively low. We also demonstrated that alternative prey can mediate predation risk to juvenile salmon. This approach is applicable to other studies focused on aquatic predator-prey interactions where direct observations of predation events are difficult to obtain. To validate our results and to enable computation of juvenile salmon predation mortality, however, diet samples of seabirds under varying environmental conditions, particularly during periods of increased or decreased plume surface areas, are required. Evidence of salmon consumption by other predator groups including marine mammals and piscivorous fish has been compiled [46, 103], but without empirical data on seabird consumption of juvenile salmon and alternative prey, the cumulative impact and relative importance of avian predators on juvenile salmon predation mortality remains unresolved.

## Supporting information

S1 Fig Distribution of juvenile coho salmon (fish km^-2^) caught in surface trawls during a) May 2010, b) May 2011, c) May 2012, d) June 2010, e) June 2011, and f) June 2012.
(TIF)

S2 Fig Distribution of yearling Chinook salmon (fish km^-2^) caught in surface trawls during a) May 2010, b) May 2011, c) May 2012, d) June 2010, e) June 2011, and f) June 2012.
(TIF)

S3 Fig Distribution of subyearling Chinook salmon (fish km^-2^) caught in surface trawls during a) May 2010, b) May 2011, c) May 2012, d) June 2010, e) June 2011, and f) June 2012.
(TIF)

S4 Fig Distribution of alternative prey (surface trawl and acoustic measurements combined, fish km^-2^) during a) May 2010, b) May 2011, c) May 2012, d) June 2010, e) June 2011, and f) June 2012.
(TIF)

S5 Fig Distribution of seabirds (common murre and sooty shearwater, birds km^-2^) during a) May 2010, b) May 2011, c) May 2012, d) June 2010, e) June 2011, and f) June 2012.
(TIF)
