# Supplementary figures and images for: Characterizing juvenile salmon predation risk during early marine residence
Source: PLoS One. 2021 Feb 19;16(2):e0247241. doi: 10.1371/journal.pone.0247241 (PMC7894896; doi:10.1371/journal.pone.0247241)

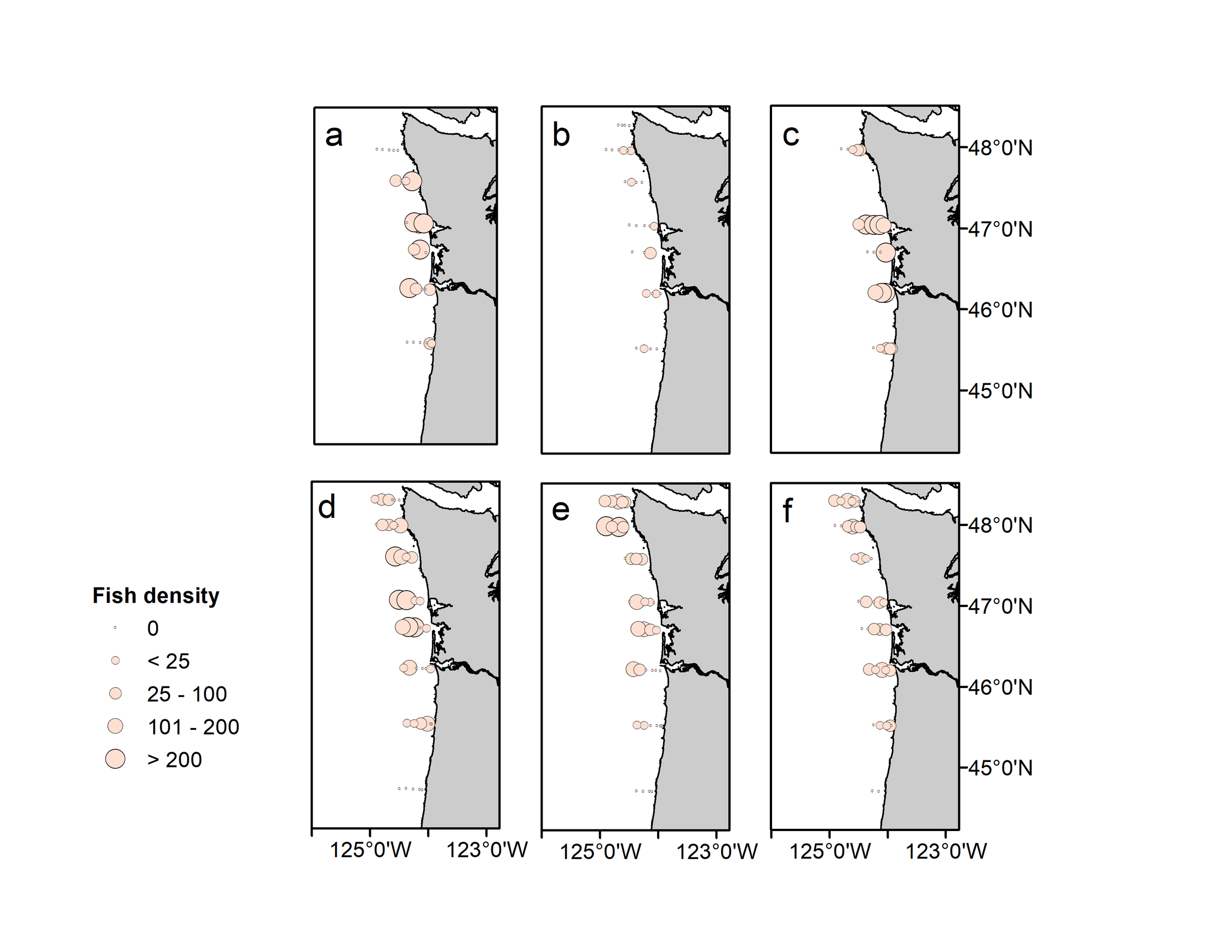

Supplement: S1 Fig — Distribution of juvenile coho salmon (fish km-2) caught in surface trawls during a) May 2010, b) May 2011, c) May 2012, d) June 2010, e) June 2011, and f) June 2012. (TIF) [file pone.0247241.s001.tif]

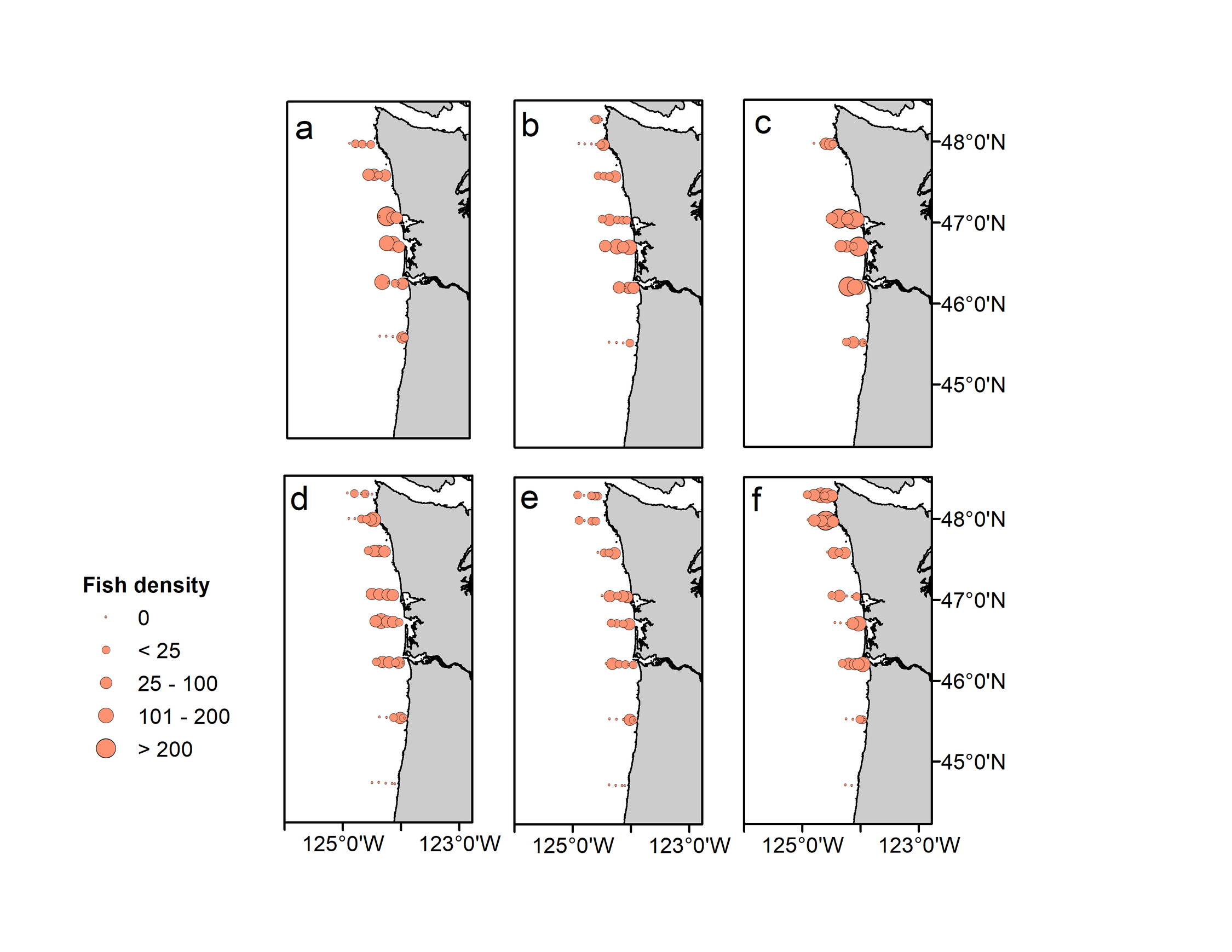

Supplement: S2 Fig — Distribution of yearling Chinook salmon (fish km-2) caught in surface trawls during a) May 2010, b) May 2011, c) May 2012, d) June 2010, e) June 2011, and f) June 2012. (TIF) [file pone.0247241.s002.tif]

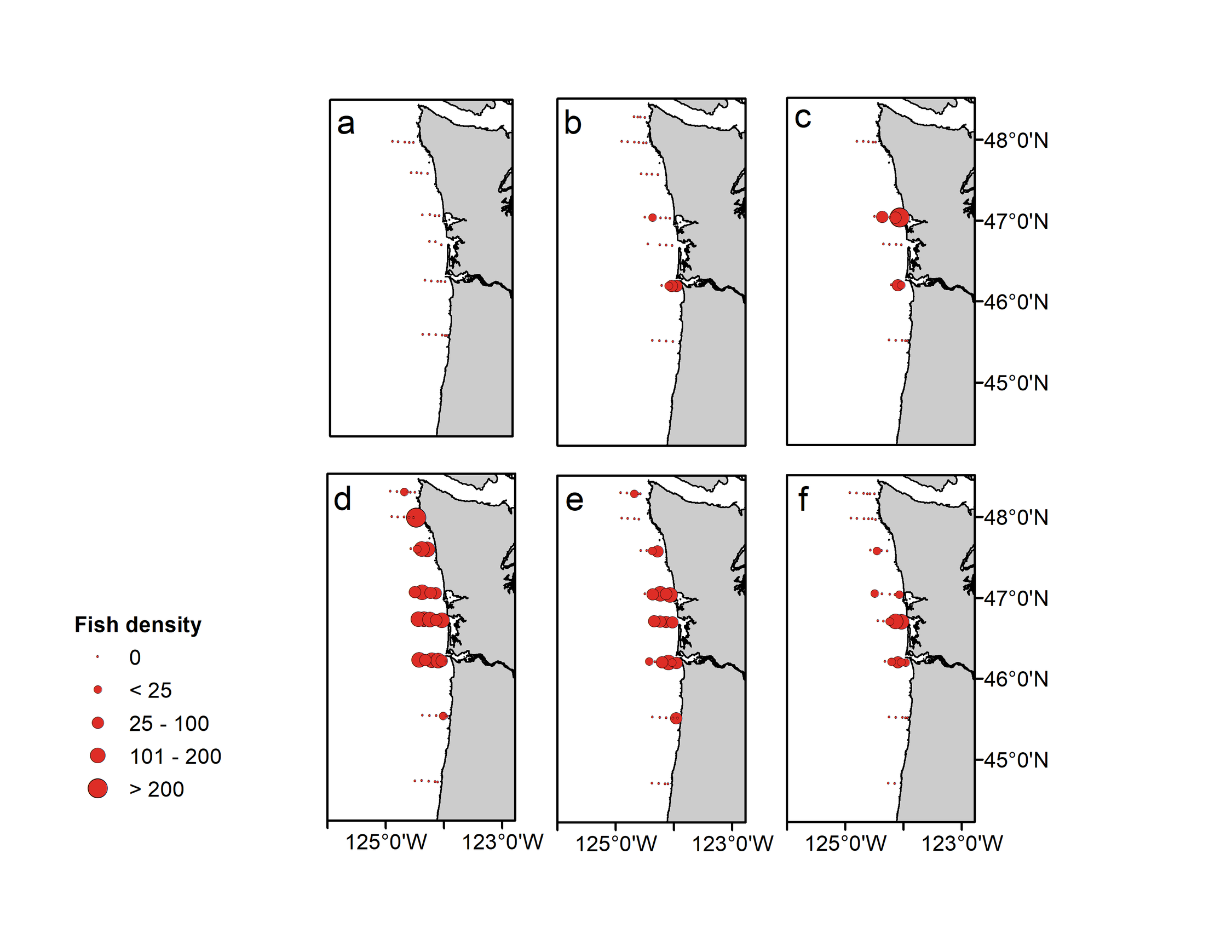

Supplement: S3 Fig — Distribution of subyearling Chinook salmon (fish km-2) caught in surface trawls during a) May 2010, b) May 2011, c) May 2012, d) June 2010, e) June 2011, and f) June 2012. (TIF) [file pone.0247241.s003.tif]

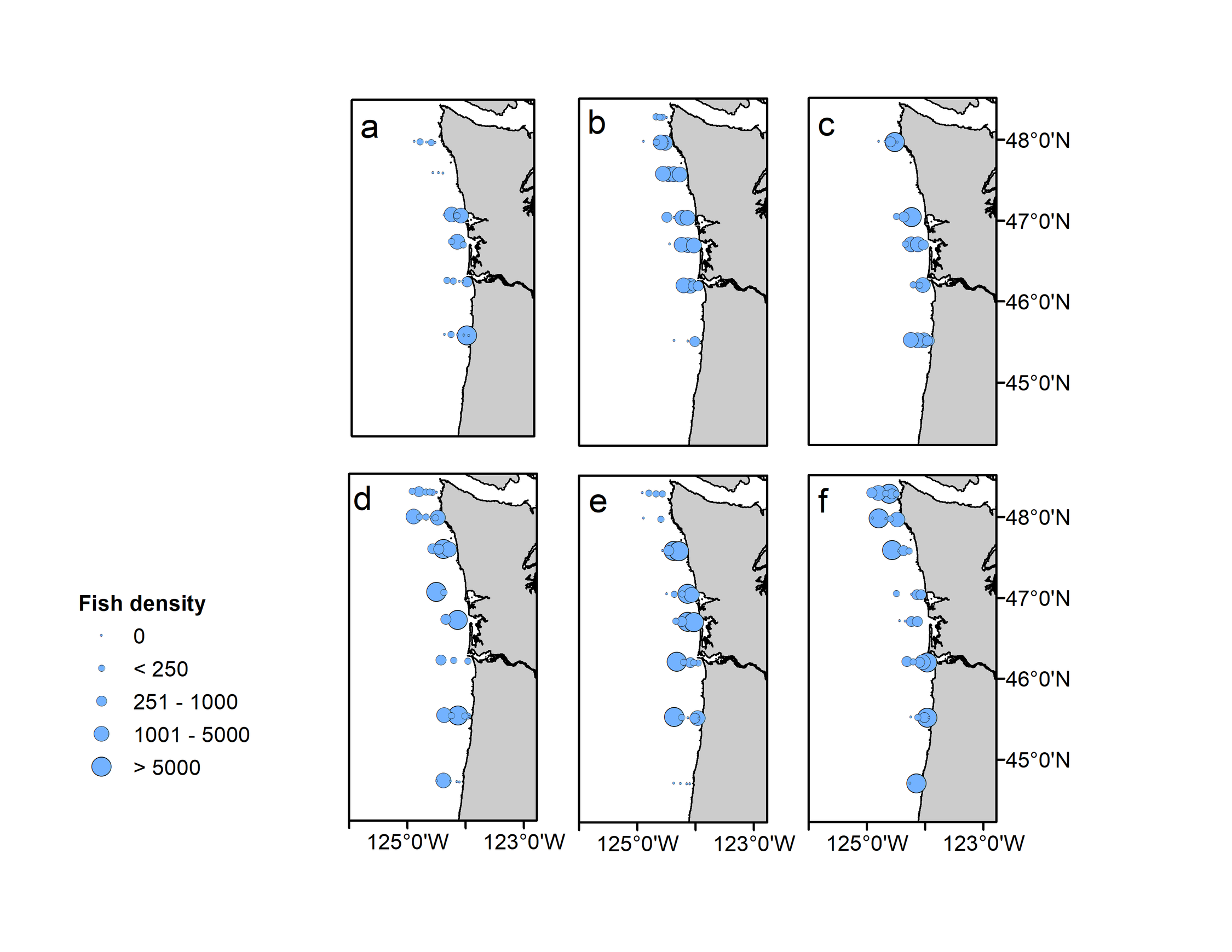

Supplement: S4 Fig — Distribution of alternative prey (surface trawl and acoustic measurements combined, fish km-2) during a) May 2010, b) May 2011, c) May 2012, d) June 2010, e) June 2011, and f) June 2012. (TIF) [file pone.0247241.s004.tif]

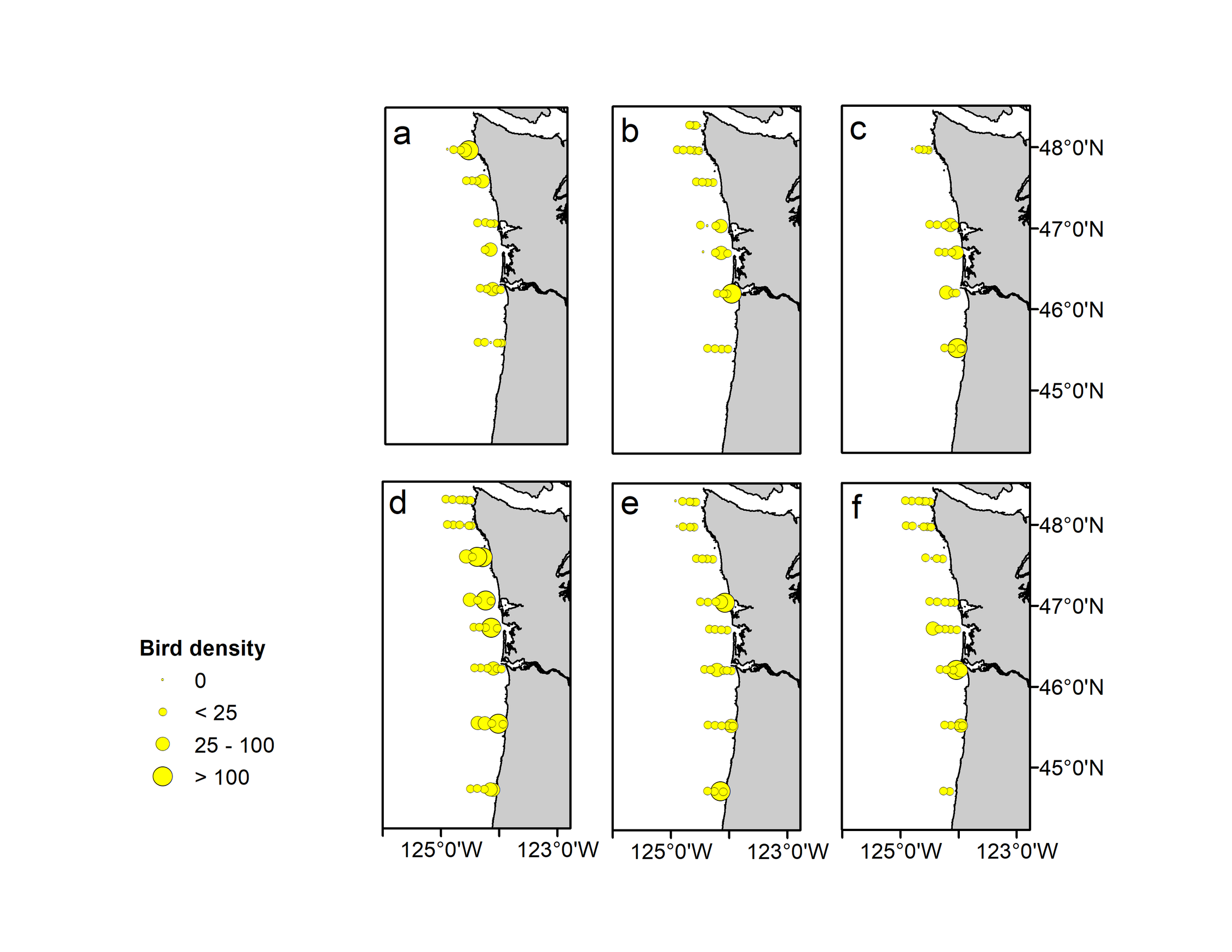

Supplement: S5 Fig — Distribution of seabirds (common murre and sooty shearwater, birds km-2) during a) May 2010, b) May 2011, c) May 2012, d) June 2010, e) June 2011, and f) June 2012. (TIF) [file pone.0247241.s005.tif]
